# Studying the impact of translational genomic research: Lessons from eMERGE

**DOI:** 10.1016/j.ajhg.2023.05.011

**Published:** 2023-06-20

**Authors:** Ellen Wright Clayton, Maureen E. Smith, Katherine C. Anderson, Wendy K. Chung, John J. Connolly, Stephanie M. Fullerton, Michelle L. McGowan, Josh F. Peterson, Cynthia A. Prows, Maya Sabatello, Ingrid A. Holm

**Affiliations:** 1 Center for Biomedical Ethics and Society, Departments of Pediatrics and Health Policy, Vanderbilt University Medical Center, Nashville, TN 37203, USA; 2 Department of Medicine, Center for Genetic Medicine, Northwestern University Feinberg School of Medicine, Chicago, IL 60611, USA; 3 Department of Medicine, Vanderbilt University Medical Center, Nashville, TN 37232, USA; 4 Departments of Pediatrics and Medicine, Columbia University, New York, NY 10032, USA; 5 Center for Applied Genomics, Children’s Hospital of Philadelphia, Philadelphia, PA 19104, USA; 6 Department of Bioethics & Humanities, University of Washington School of Medicine, Seattle, WA 98195, USA; 7 Biomedical Ethics Research Program, Department of Quantitative Health Sciences, Mayo Clinic, Rochester, MN 55905, USA; 8 Department of Women’s, Gender, and Sexuality Studies, University of Cincinnati, Cincinnati, OH 45221, USA; 9 Center for Precision Medicine, Department of Biomedical Informatics, Department of Medicine, Vanderbilt University Medical Center, Nashville, TN 37203, USA; 10 Divisions of Human Genetics and Patient Services, Cincinnati Children’s Hospital, Cincinnati, OH 45229, USA; 11 Center for Precision Medicine & Genomics, Department of Medicine, and Division of Ethics, Department of Medical Humanities & Ethics Columbia University Vagelos College of Physicians and Surgeons, NY, NY 10032, USA; 12 Division of Genetics and Genomics, Boston Children’s Hospital; Department of Pediatrics, Harvard Medical School, Boston, MA 02115, USA

**Keywords:** translational research, genomics, ethics, return of results, research design

## Abstract

Two major goals of the Electronic Medical Record and Genomics (eMERGE) Network are to learn how best to return research results to patient/participants and the clinicians who care for them and also to assess the impact of placing these results in clinical care. Yet since its inception, the Network has confronted a host of challenges in achieving these goals, many of which had ethical, legal, or social implications (ELSIs) that required consideration. Here, we share impediments we encountered in recruiting participants, returning results, and assessing their impact, all of which affected our ability to achieve the goals of eMERGE, as well as the steps we took to attempt to address these obstacles. We divide the domains in which we experienced challenges into four broad categories: (1) study design, including recruitment of more diverse groups; (2) consent; (3) returning results to participants and their health care providers (HCPs); and (4) assessment of follow-up care of participants and measuring the impact of research on participants and their families. Since most phases of eMERGE have included children as well as adults, we also address the particular ELSI posed by including pediatric populations in this research. We make specific suggestions for improving translational genomic research to ensure that future projects can effectively return results and assess their impact on patient/participants and providers if the goals of genomic-informed medicine are to be achieved.

## Teaser

This article identifies challenges in recruiting participants, returning results, and assessing their impact over four cycles of the Electronic Medical Record and Genomics (eMERGE) Network as well as steps taken to address these obstacles in order to make specific suggestions for improving translational genomic research.

## Introduction

The Electronic Medical Record and Genomics (eMERGE) Network was formed to conduct research combining genomic data with clinical data from electronic medical records (EMRs) (see web resources). The methods and tools developed through eMERGE have generated hundreds of discoveries (https://emerge-network.org/publications/) and supported the return of monogenic findings and polygenic risk scores (PRSs) to participants. From the beginning, eMERGE research projects embedded empirical and normative investigation of the ethical, legal, and social implications (ELSIs) related to the scientific use and return of clinically relevant genomic results. While the cycles of eMERGE differed in their focus, overarching lessons can be learned from this large national network (see Table 1).

**Table 1:** Phases of eMERGE

	**Focus**	**ELSI investigations**	**Participants**
eMERGE I 2007–2011	feasibility of using clinical data from EMR for genomics research	⋅ consent for genomic research ⋅ obtaining results from the EMR ⋅ privacy of genetic information ⋅ sharing genomic data in accordance with the (then) new NIH GWAS policy ⋅ community engagement with representatives of the biobank populations included in the research, as well as other stakeholders^1^^,^^2^^,^^3^^,^^4^^,^^5^^,^^6^	∼17,000 previously collected samples primarily from people of European ancestry
eMERGE II 2011–2015	continued feasibility plus pharmacogenomic testing and return of results	⋅ returning results to biobank participants not previously consented to receive results ⋅ return of research results and their clinical integration into the EMR ⋅ survey of public attitudes about consent and data sharing^7^^,^^8^^,^^9^^,^^10^^,^^11^	9,000 participants, including children
eMERGE III 2015–2020	returning actionable monogenic results to participants and providers	⋅ determining what types of results to return and how to do so ⋅ educating study participants and clinicians about genetic information and the types of results that might be returned ⋅ returning research results to children ⋅ comparing methods of return, engaging stakeholders in the return of research results	25,000 participants, including children
eMERGE IV 2020–present	returning genome informed risk assessment based on polygenic risk scores, family history, a limited number of monogenic variants, and clinical risk information to participants and providers	⋅ recruiting diverse populations ⋅ relative lack of knowledge about the predictiveness of many PRS in racial and ethnic populations who have been underrepresented in genomic research ⋅ providing to participants and their providers the information needed to understand results ⋅ engaging participants and providers in assessment of clinical and social outcomes^12^	25,000 participants, including children; cohorts comprise 70% of historically underrepresented communities in biomedical research; this is the first national research network to collect self-reported data on disability status and relationship to participation

Throughout these four cycles, the Network has confronted a host of challenges and has learned much in addressing them. Our goal is to share what we have learned and to make suggestions for improving translational genomic research. We divide the issues into four broad categories: (1) study design, including recruitment; (2) consent; (3) returning results to participants and their health care providers (HCPs); and (4) assessing follow-up care of participants and measuring the impact of research on participants and their families. Throughout, we address the ELSIs related to the inclusion of pediatric populations in the research.

## Study design

### ELSI research to inform consent and return of results occurred contemporaneously with obtaining and analyzing samples for the main study

Throughout eMERGE, ELSI studies were often embedded. In eMERGE IV, the NHGRI (National Human Genome Research Institute) felt it was important for ELSI studies to inform the processes of the main study and be conducted in year 1 prior to enrollment. Thus, the NHGRI RFA mandated that the first year be dedicated to ELSI studies designed to inform later recruitment and return of results activities. However, the timing was such that the results were obtained too late to do so. The ELSI projects at many sites involved interactions with stakeholders and local communities in investigations related to reporting PRSs, engaging diverse populations, and returning uncertain results. Although conducting ELSI studies to inform the main study was a laudable goal, the pressure to start enrolling in the main study quickly meant that processes were largely in place by the time the results of the one-year ELSI projects were becoming available, diminishing the ability of these studies to inform the cohort intervention study.

### Recruiting diverse populations through academic medical centers poses challenges

Genetic research has often failed to include adequate diversity in translational research efforts, which severely limits how well the results of these studies can be used with the general population.^13^^,^^14^ Phases I–III relied heavily on samples from sites’ existing biobank repositories, which were largely derived from individuals of European ancestry. To improve diversity of biobank participants, some sites used community engagement strategies during one or more of these phases to learn about and develop relationships with populations that may have been disenfranchised from research.^15^^,^^16^^,^^17^^,^^18^ Developing these relationships to identify and address concerns often takes years, which was not always in place for eMERGE sites.

An early but persistent challenge faced by investigators was contacting participants and ensuring follow-up.^18^^,^^19^^,^^20^ As a result, it was decided that participants in eMERGE IV must be receiving health care at the study site, both to facilitate return of research results and to be able to access medical records to assess subsequent outcomes.^7^^,^^8^ These relationships, however, do not always exist for underrepresented groups, and this requirement may limit enrollment. Nonetheless, despite this requirement, there has been success in increasing the diversity of eMERGE IV participants, often via iterative two-way communication processes with a broad and diverse group of stakeholders.^15^^,^^18^^,^^21^

### Variation among protocols posed issues for analysis

Genomics consortia have often faced challenges in harmonizing ELSI outcome measures, going back at least to the Breast Cancer Consortium in the 1990s.^22^ Variation can be attributable to the different situations and goals of study sites. Such variation, however, complicates analysis,^23^^,^^24^ as described further below, making it desirable to develop at least some uniform instruments to the extent possible.

### Including children and adults in the same study of genomic contributions to disease

Inclusion of adults and children in the same study in eMERGE II, III, and IV added further complexity. While it is critical to include individuals in research over the lifespan, children are not small adults, and using the same protocols for children and adults is often not possible due to differences in prevalent diseases, consent processes, involvement of families, and differing guidelines for clinical actionability.^25^^,^^26^^,^^27^

The first issue is that many diseases differ in the age of onset—some present only in childhood, some only in adults, and some in both. The age of onset affects decisions regarding which diseases with genetic predispositions are appropriate for asymptomatic screening. There is little clinical utility in screening adults for genetic predisposition for diseases that present predominantly in childhood (e.g., retinoblastoma, type 1 diabetes). Likewise, screening children for adult-onset disorders is viewed by many as ethically problematic, raising questions around child autonomy and open future as well as the psychosocial impacts of such information before (and if) actions on these findings can take place.^25^^,^^28^ Choosing disease and other outcomes that can combine data from children and adults needs careful consideration in the early design stages to obtain meaningful data for each population. As a result, particularly in eMERGE IV, which studies PRSs for common diseases, many of the diseases studied in adults are different from those studied in children, complicating the integration of pediatric and adult data.

The second issue is that data collection and analysis is problematic when research cohorts include both children and adults. Adults provide data about themselves. Parents, by contrast, usually provide proxy data for pediatric participants, particularly those who are young, and so may not fully reflect the children’s experience. In addition, exclusion of children’s views, while practical with young children, may inadequately respect the emerging autonomy of older children.^25^^,^^28^^,^^29^^,^^30^^,^^31^^,^^32^^,^^33^ Adolescents are an important subset of the pediatric population and frequently want to be directly engaged in return of results and express their perspectives.^34^^,^^35^^,^^36^^,^^37^ Moreover, many adolescents are capable of completing surveys themselves to reflect their own experience,^34^ but enabling them to do so requires a separate set of surveys to collect pediatric data. Information about adults and children is thus often not entirely comparable.

The third issue, specific to eMERGE IV, is that all sites are required to enroll children. Sites that primarily planned to enroll adults are finding enrollment of children challenging and thus may likely enroll few children. Requiring clinical sites to enroll children when the study team is made up primarily of adult providers with limited pediatric experience places a high burden on these sites. As a result, the pediatric sample size will likely be small, potentially reducing the impact of pediatric findings from eMERGE IV.

#### Major lessons learned in study design

• If the plan is to conduct ELSI (or other) studies to inform intervention studies, provide time to carry out and analyze the ELSI studies’ findings first so they can inform these trials.
• Enrollment of diverse populations in genetic research requires lead time for building relationships and gaining community trust prior to the design and conduct of research.
• Consider the implications of study design decisions on the enrollment of diverse populations.
• A single protocol across sites allows for comparison of many data points on return of results and their value to participants.
• Enrolling both pediatric and adult populations in the same site is complicated and needs to be clearly thought through in initial stages of study design and should be done only when site investigators have adequate expertise and infrastructure to address both populations.
• Careful consideration should be given to collecting survey and other parent-generated data in a study involving children and adults, given limitations of combining pediatric and adult data.
• Children should have their own adequately powered protocols conducted by sites that have expertise in conducting research with minors. There is also a need for ensuring that recruitment, consent approaches, and survey material are tailored to children of varying ages and the growing autonomy and capabilities of adolescents.

## Consent

In eMERGE, issues around obtaining informed consent have been pervasive. Investigators prioritized developing educational materials using plain and inclusive language, imagery, and examples that are relatable to a diverse participant audience.^1^^,^^17^^,^^36^^,^^38^^,^^39^^,^^40^ It is also the first national research network to collect self-reported data on disability status. In eMERGE I and II, investigators engaged local communities, biobank participants, healthcare providers, and other stakeholders to learn about their views on genomic medicine and its integration into healthcare.^1^^,^^2^^,^^8^^,^^41^^,^^42^^,^^43^^,^^44^ These discussions and interviews highlighted the need for careful communication and transparency about how data (including genomic data) would be used and shared. The use of audiovisual materials to complement consent and other study materials was noted to be important to address the lack of understanding of genomic information by the public.

### Identifying issues to be addressed in consent, particularly regarding return of results

In later phases of eMERGE, many individuals were specifically recruited for the studies, offering, and in some cases requiring, that individual results be returned to participants and placed in their EMR. The decision to recruit participants prospectively was motivated in part by changes in the Common Rule that now specifically require detailed disclosure to potential participants about “whether clinically relevant research results will be disclosed to subjects, and if so, under what circumstances.”^45^ This spurred much research to learn how best to obtain informed consent. At a minimum, participants need to be told what results they are likely to receive. They also need to know that the validity of their results may be dependent on what they reveal about themselves. For example, the genetic results for bone marrow transplant patients may reflect their donor’s genotype, and transgender patients will receive an interpretation congruent with their sex assigned at birth rather than their preferred gender identity. Assessing how well individuals understand what is involved in this research is an ongoing challenge.

### Broad consent and data sharing

Since the beginning of eMERGE, the Network has dealt with evolving requirements for broad consent and data sharing.^9^^,^^10^^,^^46^ Of particular note, during eMERGE II, the Consent, Education, Regulation, & Consultation (CERC) workgroup received supplemental funding from the NHGRI to learn more about public views regarding broad consent for data sharing in biobanks by surveying patients at participating institutions who were not necessarily enrolled in eMERGE itself.^9^^,^^10^^,^^46^ In this study, which involved 13,000 respondents, investigators found that approximately two-thirds of respondents were hypothetically willing to participate in research regardless of consent type or degree of data sharing. Those who identified as Black, had less education, lower income, less trust in healthcare and researchers, or more concerns about privacy were more reluctant to participate. While other studies showed that sharing of genetic data in genomic databases is generally viewed favorably by potential research participants,^38^^,^^47^^,^^48^^,^^49^ many want to be asked for consent before data are actually shared.^50^

### Why people decline to participate

Another critical issue is why individuals decline to participate. For example, some participants expressed concerns during the consent process about having genetic information from the study placed in their EMR. At times, this appeared to reflect misconceptions about the EMR. A decliner survey could have facilitated determining whether this was an issue. However, we were unable to study whether the return of results led some people to choose not to participate in eMERGE because the IRBs would not permit investigators to ask decliners about their decisions.^51^

### Parent permission and child assent

Although some urge that adolescents over 13 years of age are as capable of providing consent as adults if provided with age-appropriate material and time to reflect on the issues,^36^ the Common Rule still requires parental permission until the child turns 18 years of age. IRBs vary when it comes to age requirements for child assent and when child dissent can overrule parent permission. The need to re-consent participants who enrolled as minors and then reached the age of majority during the study in order to continue participation has presented pragmatic and ethical challenges.^52^ Occasionally, sites could not locate these individuals, or they did not respond and re-consent. This was a particular problem in eMERGE III—in a few cases, a child who enrolled at <18 years of age had an actionable finding but had turned 18 prior to return. Sites could no longer return the now legal adult’s result to the parent and without new consent to participate in the study and learn results, could not return the actionable finding to these now legally adult participants. In one case, which illustrates this complexity, a known pathogenic variant in *RYR1* was reported by the central laboratory on a pediatric biobank participant who enrolled as a child but who turned 18 before results were available for return. Bound by IRB protocol and the Common Rule, the adolescent had to first consent to the biobank as an adult and consent to recontact for results before the site could begin the process of contacting the adolescent to communicate the eMERGE result and to get permission to place the result in the clinical record. Fortunately, all of the above occurred, and a result with critical clinical ramifications was returned to the adolescent before the study concluded.

### Follow up when participants had died

Another issue arose when participants in eMERGE III died before an actionable finding of an autosomal-dominant disease was returned.^53^^,^^54^ In these cases, there were implications for family members. Without knowing the deceased participant’s preferences in this regard, sites were unclear as to how to proceed, highlighting the need to address return of results upon participant death in the consent form. This has been addressed in eMERGE IV at the time of enrollment by having the participant indicate someone to receive results in the case of a participant’s death.^55^

#### Major lessons learned in consent

• Potential participants should be informed what results they may receive and when they may receive them.
• Engage diverse communities in genomic research and find multiple methods for communicating about the research and its potential findings.
• If IRBs allow, implement a decliner survey to understand why some individuals do not want to participate in the research.
• A robust plan to re-consent pediatric participants once they reach the age of majority and discuss the need to do so at enrollment of older minors is essential. Alternatively, if feasible and the time between data collection and return of results is short (e.g., <12 months), consider not enrolling children who are likely to reach the age of majority before results are returned.
• Consent processes should invite participants to nominate someone else to receive their results in the event of their death.
• Transparency and communication with participants, especially around data use and sharing, is vital to gain trust, even if it means some individuals do not enroll due to concerns about data sharing or EMR return.

## Returning results to participants and their health care providers

One of the major lessons of eMERGE was that returning results was challenging for both participants and their health care providers (HCPs), each in their own ways.

### Returning results to participants

Shortly after its inception, eMERGE investigators began to consider returning individual research results to participants,^56^ an issue that has persisted throughout the course of eMERGE.^8^^,^^12^^,^^20^^,^^24^^,^^27^^,^^35^^,^^57^^,^^58^^,^^59^ At first these included incidental findings discovered during quality control checks, such as sex chromosome variants.^19^ Over the course of eMERGE, interest in returning individual research results to participants grew. Social forces included investigator perceived obligations,^60^ participant expectation,^61^ the production of results that are increasingly used in clinical care, and the movement to provide data access in the clinic embodied in part by the Health Information Technology for Economic and Clinical Health Act (HITECH). The NHGRI, which funds eMERGE and prioritizes studies that inform genomic medicine, required the return of various classes of genomic risk information to participants to assess their utility for patient care. These results included pharmacogenomics variants (phase II), Mendelian disease risk (phase III), and most recently, PRSs for common chronic diseases as well as a limited number of Mendelian risks (phase IV).

### Consent issues

Since eMERGE phases I and II primarily used samples and data from biobank participants who had not specifically enrolled in eMERGE, these individuals had not provided consent to receive results from the eMERGE study. This led to much debate and research about the circumstances under which it might be acceptable to recontact participants with actionable results, particularly when they had not been initially asked for such consent.^19^ The Network ultimately concluded that biobank participants could receive results only if they had agreed to be recontacted about results when they enrolled in the biobank. This required eMERGE investigators to recontact participants who had a result to return and to provide the results only to those who responded and agreed to learn their results.^19^ Most sites in eMERGE III enrolled participants prospectively and obtained consent at the time of recruitment for the study, which included the return of results.

### Variability in what results were returned

A complication in eMERGE III was that the results returned varied across sites.^62^ Although all sites returned pathogenic and likely pathogenic variants in conditions that were considered clinically “actionable” according to ACMG (American College of Medical Genetics and Genomics) guidelines,^24^^,^^58^^,^^63^ sites could choose among an additional 50 or so genes on the eMERGE III list to return.^58^ In addition, one site returned variants of uncertain significance (VUSs) for one disorder. Negative results were returned to participants at some sites,^64^^,^^65^^,^^66^ and one site returned carrier status.^35^ Sites also varied in whether they re-interpreted results over time in light of new information about clinical significance.^67^^,^^68^ This variability complicated the study of outcomes. In order to avoid these issues, eMERGE IV is generating and returning PRSs for several common diseases using a central IRB and a single Network-wide protocol. This assures that all participants are prospectively enrolled, provide a sample for DNA collection, and receive a uniform genome informed risk assessment (GIRA) report that includes PRSs, although the diseases assessed in the report depend on age (one report for those <18 years with four PRSs and one for those 18 years and older with 10 PRSs).^12^

### Variability in how results were returned

How results were returned varied over time and across the sites.^20^^,^^24^^,^^67^^,^^69^^,^^70^^,^^71^^,^^72^^,^^73^ In eMERGE III, which had a robust return of results component, results were sent to participants by mail^42^ or provided in the participants’ patient portal, and increasingly were placed in participants’ EMR. At most sites, genetic counselors and/or other clinicians affiliated with the study reached out to participants to provide results that were deemed to be clinically pertinent, although practices varied across sites. Despite the desire of study staff to counsel participants with actionable results directly, less than half of participants pursued counseling.^74^^,^^75^ While participants were more likely to receive counseling if they were required to or specifically invited as part of the protocol, even then, uptake was by no means complete.^24^ Moreover, only about one-third of participants shared positive test results with family members, and even among those who received genetic counseling, only half shared the results with a family member.^76^ In addition, due to delays in analysis and a long elapse of time between enrollment and the availability of results, it was at times difficult to locate participants who had agreed to receive results.^63^ This ultimately led investigators to adopt a general practice of sending certified letters with actionable results to participants who could not otherwise be found and inviting contact.

There was much greater variation across sites in how negative results were returned.^24^^,^^66^ While recipients who were informed that they did not have actionable results were generally content with the process,^77^ some of the respondents incorrectly felt that they had less or no residual risk.^74^^,^^77^

### Situations that limited the return of results

As noted above, pediatric sites were not able to return results to children who reached the age of majority if they could not re-contact them. And if the participant died after enrolling in the study, results could not be returned.

#### Major lessons learned about returning results to participants

• Prospective enrollment with informed consent that includes return of results and data sharing is preferred.
• Shorten timelines from enrollment to return of results as much as possible, and if the timeline is long, consider methods to keep in contact with participants and facilitate updating of contact information.
• More planned intentional study interactions with participants about result return, including whether, when, and how results may be offered, are warranted to ensure that they understand the information.
• Many participants do not seek counseling or share information with their families after receiving actionable results, suggesting that more education is needed or that the results may be less salient than expected for participants.

### Returning results to HCPs

A major goal of eMERGE III and IV has been to study how genomic screening results were used by HCPs and integrated into clinical care. To do so, results are placed in the participants’ EMR, and HCPs are notified of the result. These participants’ research results were, by definition, unsolicited by the participants’ HCPs as the latter did not order the tests. This raised questions about whether participants’ HCPs would assume responsibility for providing appropriate follow-up themselves or by referral. In eMERGE III, the RFA (request for application) did not stipulate that sites study the impact on HCPs, and thus most sites did not include these studies in their projects; only VUMC (Vanderbilt University Medical Center) planned and conducted an HCP interview study.^59^^,^^78^ Once the Network study was underway, investigators felt it to be important to explore this topic. As a result, a separate grant was funded across all clinical sites using interviews and a survey to explore this issue.^23^ These studies revealed that while many of the participants’ HCPs felt responsible for follow up, their level of comfort with doing so was often low. They often wanted more decision support and access to pertinent specialists.^23^^,^^59^ Concerns by primary care providers (PCPs) about their ability to provide adequate follow-up gave further weight to the growing practice of eMERGE investigators’ assuming responsibility for directly contacting participants found to be at elevated risk to offer genetic counseling. This development, however, means that eMERGE provides less practical insight into how genomic tests can be effectively incorporated into primary care.

#### Major lessons learned in the return of results process to HCPs

• Funders should provide resources for investigators to study the impact of return of results on HCPs up front, as this is an important part of the implementation of genomic medicine.
• Given that there may be few HCPs per site receiving results in some cases, studies should be Network-wide.
• PCPs’ capacity and expertise in managing patients’ care should be optimized in translational studies by involving PCPs in planning and providing adequate support, which includes providing clear notification about the results and deploying educational resources, such as decision support mechanisms.
• In many cases in genetic research, it may be best if the investigator team provides initial results and counseling to participants.

## Assessing follow-up care of participants and impact of research on participants and their families

Another major goal of the eMERGE Network is to assess the medical outcomes and impact of returning genomic results on participants and providers. In early stages of eMERGE, this was accomplished through review of medical records in a standardized manner across the Network.^3^^,^^79^ Challenges arose here as well.

### Assessing outcomes in a limited time frame

One of the most challenging issues has been the limited time frame of each phase (4–5 years) due to the constraints of National Institutes of Health (NIH) funding mechanisms. This time limitation curtails the ability to assess outcomes effectively, particularly as predictive and predisposition testing becomes more prominent. Analyzing and returning results frequently encountered prolonged delays, further shortening the time available to assess the medical and psychosocial outcomes of providing genetic information to participants. The challenge of a short time frame will be especially salient for the current eMERGE IV study, where PRSs are being implemented for more common diseases that often manifest at later ages, such as obesity, diabetes, and cardiovascular diseases. While eMERGE IV is measuring HCP recommendations, which may include lifestyle and dietary changes, as well as tests to screen for early signs of diseases and other changes in clinical care aimed at early detection or prevention, these are long-term processes that often occur over years. Measuring clinical outcomes for conditions of this type takes many years of follow up to detect incremental physiologic changes. To complicate matters further, study timing in eMERGE, particularly in phases II–IV, included a planning phase to develop and gain agreement on protocols and infrastructure, regulatory approvals, recruitment, analysis, validation of results, and return of results. These processes take time and thus cut into the follow-up period after the return of results to, at best, no more than 3 years. For those who are enrolled toward the end of recruitment, only a year or less is possible for follow-up after return of results. These time constraints limit what can be learned from translational research efforts. If the study cannot adequately achieve the goal of assessing the impact of returning genomic information, the studies cannot fully meet their obligation to the participants who enrolled in the research, as well as those who fund it.

The lack of long-term follow-up is a limitation of much government-funded research. Although there are exceptions (e.g., the Nurses’ Health Study, Framingham Heart Study, and Jackson Heart Study), the vast majority of studies are funded on 4- to 5-year cycles. The NIH and other funders should consider alternative structures for cohort studies that include study interventions that take years to implement in order to assess their impact on health outcomes. For multi-phase studies such as eMERGE, for example, investigators could be encouraged to revisit outcomes in participants from prior research cycles, extending the window of potential follow-up. This assessment could weigh the costs of follow-up against the value of learning about the long-term benefits of preventive measures and early detection in real-world healthcare.

### Assessing participant-centered outcomes

The short time horizon for participant follow-up after result return compromised assessment of whether results had any direct impact on participants’ lifestyle or healthcare decision-making and limited assessment of long-term health outcomes. In eMERGE III, the collection of medical outcomes through the EMR was standardized across the Network, but participant surveys were not, resulting in variation in survey instruments across sites.^23^ Recognizing that a small number of participants at each site would receive a positive result, the Network attempted to harmonize the participant survey data collected across sites in order to increase the power of these studies, leading to a publication that assessed the experience of the 1,444 participants (out of >25,000 or ∼6%) who received a pathogenic variant^24^ and another on the familial implications of return of results.^23^ This, however, was a challenge as, despite efforts to ask a limited set of questions on all site surveys, the sites did not always use the same language. In addition, as mentioned above, the data from pediatric sites could not be used as parents completed the survey for their child or adolescents completed surveys for themselves and so were not comparable with the data from adult sites. Harmonizing the data upfront by having all sites use the same survey, as in eMERGE IV, helps to ameliorate this issue, although issues with pediatrics remain.

### Variation in access to follow-up care

Another issue is that the investigators in eMERGE generally returned only results with potential to alter the care of participants themselves, i.e., results suggesting the need for further diagnostic workup or initiation of recommended surveillance and/or prophylaxis in the follow-up care. In the early stages of eMERGE, the strategies used for recruitment meant that almost all participants had insurance coverage and access to specialty care. As efforts were made to increase the diversity of participants by recruiting in federally qualified health centers and by returning results such as PRSs (ongoing in eMERGE IV) whose implications were not yet well accepted, ensuring access to recommended follow-up care became more challenging. In eMERGE IV, research funding may be provided for some follow-up evaluations for participants who lack insurance coverage although long-term assessment of the impact of results on health behavior may be limited.

### Assessing outcomes in children

As discussed above, eMERGE prioritizes the inclusion of both children and adults in the same study. However, there are many more adult sites than pediatric sites, and therefore many more adults enrolled than children. This structure compromises the power to assess outcomes in children as a sub-cohort, especially in eMERGE IV where risks for only four conditions are studied in children compared with ten disorders in adults.

#### Major lessons learned in assessing the follow-up care of participants

• Either more time is needed for follow up to assess the impact of return of results, or endpoints need to be defined in ways that are measurable within the time frame allowed.
• Funders should consider whether providing funds to collect follow-up data from earlier research cycles, particularly for sites that have participated over time, would have sufficient yield.
• Funders should provide resources for investigators to study the long-term impact of return of results on participants through surveys and/or interviews. Better understanding of the impact of results on health behaviors and the barriers experienced by participants is an important part of the implementation of genomic medicine.
• If children and adults are included in the same study, data and outcomes should be sufficiently similar between these groups to allow the data to be aggregated.
• Wherever possible, central elements of data collection, including follow-up care following return of results, should be identical across sites.
• Given the fragmented and uneven healthcare delivery system in the United States, funders should provide resources for follow-up care for uninsured and underinsured participants to mitigate cost-related barriers to engaging diverse communities in translational genomic research.

## Blurring the lines

One of the most important challenges investigators face in eMERGE is the fact that clinical translational research programs like these inherently blur the line between research and clinical care.^57^^,^^63^^,^^80^^,^^81^ Unlike clinical care, the goals of research go beyond the interests of the individual affected individual and seek, at least in part, to make new discoveries and to understand the clinical implications of specific research practices. Research studies such as eMERGE, in particular, will continue to identify sources of uncertainty that at times differ from the clinical setting.

These differences between research and clinical care can challenge researchers, participants, and participants’ healthcare providers. The extent to which results revealed through rapid scientific discovery of genetic variants, including GWASs (eMERGE I–III), panel genetic testing (eMERGE II–IV), and PRS assessment (eMERGE IV), are well understood is not consistent. This variability is both analytically separate from and yet inextricably intertwined with studying how best to return results to participants—an issue that has been required in various ways in eMERGE II–IV. For example, eMERGE participants sometimes received research results that are not accepted as the standard of care in clinical practice (e.g., PRSs for cancer) but were nonetheless meant to be used clinically to assess outcomes. The return of information of uncertain import in translational research has important implications for participants who are also patients of healthcare systems. A major topic of ongoing vigorous academic and public discussion is the propriety and desirability of returning research results, including results from genomic research, with some arguing that returning research results is desirable and even required,^82^^,^^83^^,^^84^ while others urge that this can be problematic.^56^^,^^85^^,^^86^^,^^87^ All these developments, moreover, occurred in the context of significant federal regulatory changes for informed consent, return of research results, institutional review board (IRB) requirements,^45^ patient access to medical records (HITECH), and clinical and genomic data sharing.

## Summary points

The eMERGE Network has generated hundreds of scientific publications and tools and returned genomic results to tens of thousands of participants and their providers. Clearly, more long-term, large-scale projects such as these are essential to reap the full benefit of genomics, but they could provide even greater value to translational genomic medicine efforts by renewed attention to ELSI lessons. Determining whether these scientific advances will improve outcomes requires attention to participant recruitment and consent, effective strategies to ensure that clinicians and participants receive genomic information and are ready to act upon it (including by assuring that barriers for behavioral changes are removed), and the time to assess the impact of return. The goal of this article was to identify some of the ethical and logistical challenges in addressing these issues in eMERGE, many of which are inherent in translational research as compared with settled clinical practice and share some strategies to overcome them. This critical evaluation of what we have learned over the 16 years of ELSI investigations reveals a mix of findings suggesting areas for improvement and learning opportunities for genomic investigators.

Some findings that are most critical to improving the translation of genomic medicine into the healthcare setting include continued meaningful engagement of stakeholder groups in research and improvement in interactions with healthcare providers. Not only must a diverse population of participants be engaged with genomic research to fully understand the effect of genomic medicine on people in the US, but meaningful engagement must be long-term and include the planning of research through to the collection of outcomes. This can only be accomplished if healthcare providers, especially PCPs, are also engaged in the research.

Following recommendations for return of genetic results to participants in research, including providing all options, i.e., type of results desired, contact in event of death, follow-up on recommendations, etc., can be labor intensive. This is particularly true when no long-term relationship exists between participant and researcher. The ideal return involves the PCP for long-term outcomes and to reinforce genomic findings over time with the patient/participant and their family, but this requires more inclusive research paradigms with buy-in and support.

A critical and overarching component of improving genomic medicine rests with the investigators and funders in recognizing the importance of study design. The experience in the 3+ phases of eMERGE have made clear the necessity of a number of considerations: allowing adequate time for meaningful community engagement that can be employed in study design, protocols that allow both children and adults to be valued participants, and timelines outside of the usual 4- to 5-year consortium windows to allow for both community input into study design as well as follow up and outcomes.

## Web resources

Framingham Heart Study, https://www.framinghamheartstudy.org/
History of eMERGE, https://www.genome.gov/Funded-Programs-Projects/Electronic-Medical-Records-and-Genomics-Network-eMERGE#history
HITECH (Health Information Technology for Economic and Clinical Health Act), https://www.hhs.gov/sites/default/files/ocr/privacy/hipaa/understanding/coveredentities/hitechact.pdf
Jackson Heart Study, https://www.jacksonheartstudy.org/
NIH Data Management and Sharing Policy, https://sharing.nih.gov/data-management-and-sharing-policy
Nurses' Health Study, https://nurseshealthstudy.org/
